# Compliance, practices, and attitudes towards VTIs (Vehicle Technical Inspections) in Spain: What prevents Spanish drivers from checking up their cars?

**DOI:** 10.1371/journal.pone.0254823

**Published:** 2021-07-19

**Authors:** Francisco Alonso, Sergio A. Useche, Javier Gene-Morales, Cristina Esteban

**Affiliations:** 1 DATS (Development and Advising in Traffic Safety) Research Group, INTRAS (Research Institute on Traffic and Road Safety), University of Valencia, Valencia, Spain; 2 Faculty of Psychology, University of Valencia, Valencia, Spain; 3 PHES (Prevention and Health in Exercise and Sport) Research Group, University of Valencia, Valencia, Spain; Monash University, AUSTRALIA

## Abstract

**Objective:**

Mechanical conditions of vehicles may play a determinant role in driving safety, the reason why vehicle periodical technical inspections (VTIs) are mandatory in many countries. However, the high number of drivers sanctioned for not complying with this regulation is surprisingly high, and there is not much evidence on what kind(s) of motives may explain this concerning panorama. This study aimed to identify the aspects that modulate the relationship between complying (or not) with VTI’s standards in a nationwide sample of Spanish drivers. The study design also addressed the drivers’ awareness regarding different risky behaviors while driving, depending on their sex and their crash record.

**Methods:**

1,100 Spanish drivers completed a survey on the aforementioned issues. An analysis of variance (ANOVA) with Bonferroni post-hoc adjustment was conducted to assess significant differences (p<0.05) in the study variables.

**Results:**

Most of the surveyed drivers (99.18%) reported that they always comply with VTI’s requirements. The main reasons to comply were related to compliance with traffic regulation and fear of penalties, while the reasons attributed to its incompliance are, instead, stated as involuntary.

**Conclusion:**

The findings of this study support the idea that more actions are needed to increase drivers’ awareness of the relevance of VTIs for road safety, as well as warning them about the dangers of neglecting vehicle checking beyond merely punishing measures. For this reason and given the greater prevalence of the issue among younger segments of the driving population, it is suggested that more emphasis on the matter could be made during novice driver’s training.

## Introduction

Traffic road safety has improved in Spain since 2003, to the extent that (partly linked to the recent COVID-19 pandemic), road crash-related fatalities have decreased by about 50% during the last year [1–3]. However, traffic crashes still constitute a huge problem of public health, and every victim counts for taking further actions aimed at enhancing crash prevention [2–4]. Previous studies suggest that public awareness and law enforcement, in addition to the development of positive attitudes towards road safety-related regulations, are required to address this issue [5–8].

Among other potential hazards, the main causes involved in road crashes are classified in order of importance as, respectively, the human factor, vehicles technical systems malfunctioning, and road or environmental factors [9]. Within the first group, behaviors such as excessive speeding [10, 11], not keeping a safe distance, showing aggressive signs or gestures [12], smoking [13] or drinking [14, 15] while driving, not wearing the seat belt [16], or not having an insurance [17] had been identified by previous research.

While the most addressed causes in the literature are those related to human behaviors [18], mechanical conditions of motor vehicles are also highly associated with road-crash mortality rates [1, 19]. Between 5 and 8% of the vehicles involved in fatal crashes in Spain lacked a proper vehicle technical inspection (VTI) [20]. That impact could be reduced if adequate improvements to the roadworthiness testing system were put in place [21]. This technical revision is a legal measure of both prevention and maintenance, which must guarantee that a vehicle complies with the minimum safety standards [22]. In this way, the mandatory inspection of motor vehicles is designed not only to reduce the economic losses derived from potentially avoidable traffic crashes but also to enhance the protection of lives within the road network [23]. However, road safety is multifactorial, and it is difficult to isolate the individual role of vehicle inspection in the prevention of road crashes [23].

Legal regulation in terms of vehicle technical inspection (VTI) in the European Union is ruled by the Directive 2014/45/EU of the European Parliament and of the Council of 3 April 2014 on periodic roadworthiness tests for motor vehicles and their trailers and repealing Directive 2009/40/EC [21]. As written in the aforementioned document, vehicles used on public roads are required to be roadworthy when they are used, and member states should be empowered to carry out roadworthiness tests. Thus, Spanish authorities are responsible for complying with the European normative through the national law [24]. In the light of these aforementioned facts, it proves necessary to generate knowledge about the Spanish driver’s awareness and compliance with the VTI’s standards and other driving behaviors.

### Objectives

The objective of this study was to identify drivers who comply or do not comply with VTI’s standards in a nationwide sample of Spanish drivers and to gather data on the reasons to comply or not with VTI’s standards, perceptions about the sanctions, and behavioral changes in case of having received a sanction. In addition, the methods presented in this research aimed to examine the awareness of the drivers concerning different risky behaviors while driving, depending on their sex and their crash record.

Based on the literature review, we expect to find a major number of drivers complying with VTI and a change of behavior in this regard for the drivers who have received a sanction due to driving with an outdated VTI.

## Materials and methods

### Sample

The data were collected from a full sample of 1,100 Spanish drivers with ages from 14 to 88 years old ($\bar{x}$ = 42.45, SD = 14.72); 678 of which were men (61.6%) and 422 women (38.4%), representing a margin of error for the general information of ± 3 with a confidence interval of 95% and a level of significance of 0.05. In terms of age, the percentage distribution was proportional to the Spanish General Census of Drivers [25]. The most represented age groups were those between 30 and 64 years old, and the less represented the ones aged between 14 and 17 (corresponding in most cases to very young drivers of small two-wheeled vehicles). Gender distribution is closely related to age; the older the sample section is, the more the proportion of women in it decreases [25]. From age 45, the percentage of women is remarkably reduced, as it happens in the general driving population. Table 1 shows the distribution of the sample in comparison with the census of Spanish drivers, which was provided by the General Directorate of Traffic (DGT; National Traffic Authority) for the last year available (2018).

**Table 1:** Distribution of drivers’ census and study sample based on age groups.

Age	Census (n° of drivers)	Distribution (%)	Sample (n° of drivers)	Distribution (%)
14–17	45,399	0.17	13	1.18
18–24	1,634,963	6.09	106	9.64
25–29	1,877,850	6.99	141	12.82
30–44	8,411,664	31.33	418	38.00
45–64	10,704,042	39.87	331	30.09
over 65	4,175,612	15.55	91	8.27
**Total**	**26,849,530**	**100**	**1.100**	**100**

*Notes*: Census data distribution extracted from DGT (Directorate-General of Traffic). (2019). [General census of drivers of the year 2018]. [Accessed 12-03-2020]. Retrieved from: http://www.dgt.es/es/seguridad-vial/estadisticas-e-indicadores/censo-conductores/tablas-estadisticas/2018/.

### Design, procedure, and instruments

For this cross-sectional, self-reported-based study, Spanish-speaking drivers completed a semi-structured telephone interview. Participants were invited to take part in the research through simple random sampling (SRS). The questionnaire was designed and applied to ensure the anonymity of its participants. We emphasized the existing laws on data protection and the fact that the gathered information would only be used for statistical and research purposes; all this was made clear to participants before the beginning of the telephone survey. A statement of informed consent was verbally checked and accepted by participants as a requirement for their voluntary participation in the survey. The average duration of the interviews was 10 minutes, with some variability due to the individual differences of each participant and situation. The importance of answering honestly to all the questions was highlighted, as well as the non-existence of wrong or right answers, to minimize the impact of a potential bias related to self-reported data. To read more about methodological limitations inherent to self-report methods, please see section “study limitations and future research”. As it might introduce very specific (and potentially confounding) conditions and differential regulations in regard to VTIs, no professional drivers were included in the study.

The questionnaire was administrated in Spanish and consisted of different sections:

The first part inquired about individual and demographic variables, such as age, gender, region of residence; it also included a brief questionnaire about driving history, including traffic crashes suffered while driving.

As for the second part, drivers were asked if they had ever driven without VTI. According to their first answer, subjects were openly inquired about the reasons for driving with or without VTI. Then, subjects answered if they found this behavior sanctionable by law, the possible answers being “yes” or “no”. Also, participants had to point out the kind of sanction they found appropriate to punish this behavior (“economic penalty”, “prison” or “temporary or permanent suspension of the driving license”), potential answers being “yes” or “no” for each sanction.

At this point, drivers were asked to evaluate the hardness of the current penalties, according to the following options: “excessive”, “adequate” or “scarce”. To end this section, and in case the drivers had received any penalties for driving without VTI, they were asked whether being sanctioned modified their behavior or not.

After completing the questions about VTI behaviors and attitudes, drivers’ crash risk perception of the following driving misbehaviors was assessed: “driving after drinking alcohol”, “driving without insurance”, “driving without having passed the VTI”, “not using seat belt”, “speeding”, “inappropriate speed to traffic conditions”, “not keeping safety distance”, “smoking while driving”, and “shout or insult while driving”; the risk was evaluated from 0 (minimum) to 10 (maximum). They were also requested to evaluate (from 0 to 10) if these behaviors should be punished. Finally, drivers were asked about the perceived probability of being sanctioned, out of ten occasions of performing this behavior.

### Ethics

The Research Ethics Committee at the Research Institute on Traffic and Road safety at the University of Valencia granted permission to perform this study, certifying that it responded to the general ethical principles stated in the Declaration of Helsinki, as required for the case of research using human subjects, including the case of underaged ones, given the minimum risk level the study setting represented to potential partakers (IRB approval number HE0001021118). Also, a verbal Informed Consent Statement containing ethical principles and data treatment details was used, explaining the objective of the study, the mean duration of the survey, the treatment of the personal data, and the voluntary participation. This information had always been provided to the participants before they completed the questionnaire. Personal and/or confidential data were not used, and the participation was anonymous, implying no potential risks for the integrity of our participants.

### Statistical analyses (data processing)

Once the data were obtained, the relevant statistical analyses were carried out by means of the Statistical Package for Social Sciences (Version 26.0; IBM Corp., Armonk, NY). To compare mean values, a one-way ANOVA (analysis of variance) and post-hoc test (Bonferroni) with gender and traffic crashes as the between-subject factor were used. Post-hoc test significance was evaluated after Bonferroni correction [26, 27], being the significance level for this study uniformly established at *p* < 0.05.

## Results

**Fig 1:**
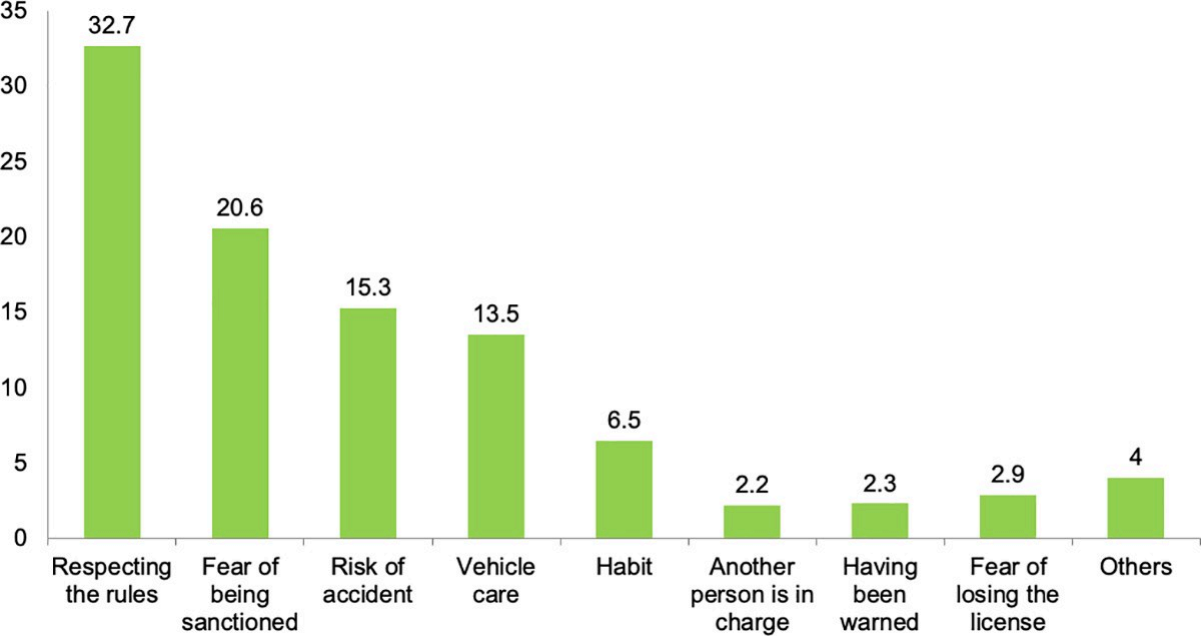
Driver’s reasons to comply with vehicle technical inspection’s (VTI) standards.

**Fig 2:**
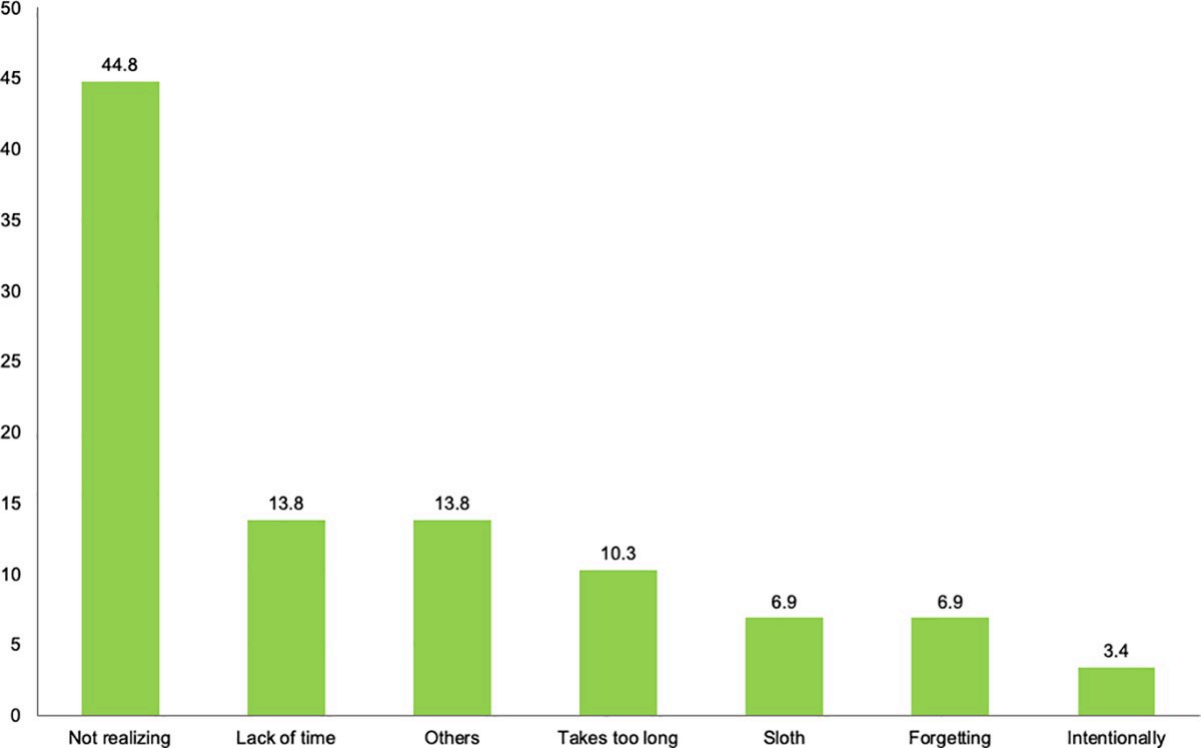
Driver’s reasons to not comply with vehicle technical inspection’s (VTI) standards.

While almost all the surveyed drivers (96.9%) self-reported that they have always driven complying with VTI’s standards and never been sanctioned, 29 of the respondents self-reported having received a sanction due to driving with a non-valid or outdated VTI. Driver’s reasons to comply or to not comply with VTI’s standards are presented in Figs 1 and 2, respectively.

**Fig 3:**
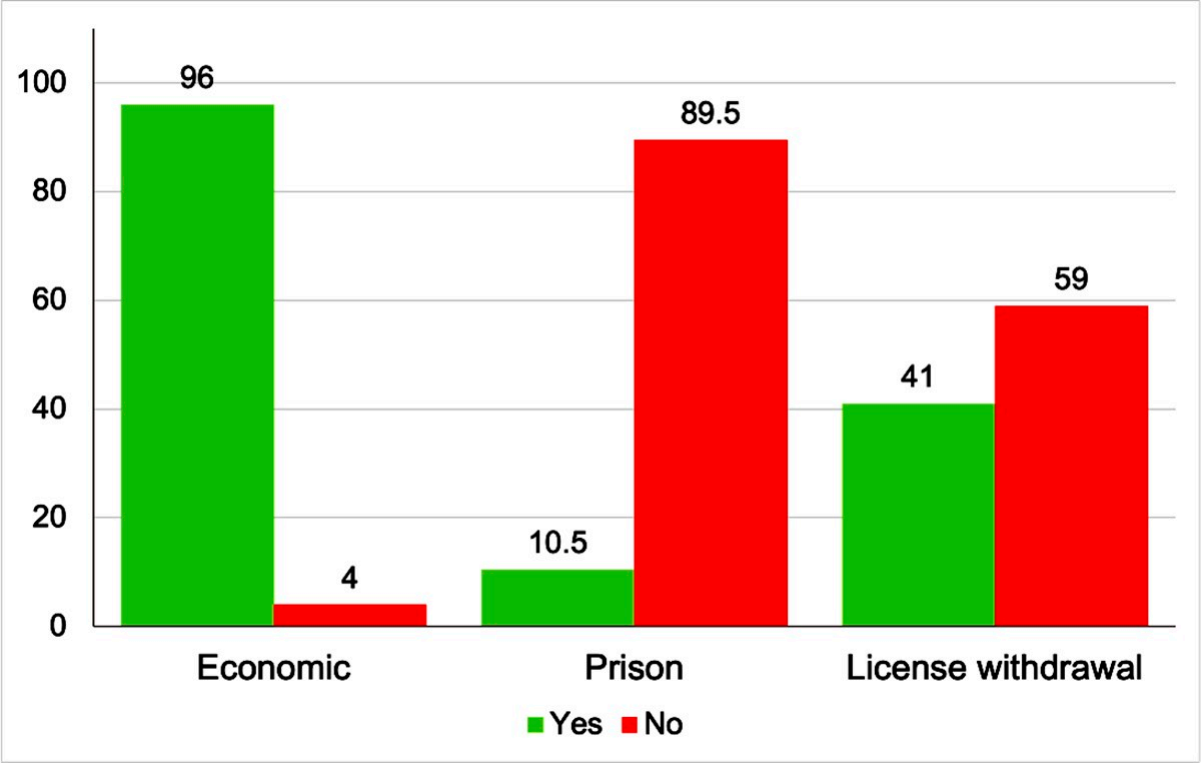
Type of sanction considered in case of VTI certificate infraction.

Almost 87% of the subjects pointed out that driving with an outdated VTI certificate constitutes sanctionable behavior. Results regarding the type of sanction that drivers found appropriate to punish non-VTI driving are shown in Fig 3.

Of the respondents who had been sanctioned due to not complying with VTI’s standards, 55.2% consider that the sanction received was excessive; 41.4% consider it appropriate; and 3.4% state that it was relatively scarce. Among the drivers who had been sanctioned due to driving with an outdated VTI, 82.8% self-reported not having driven again without complying with VTI’s standards due to the penalty, while 17.2% continued driving without a valid VTI.

**Fig 4:**
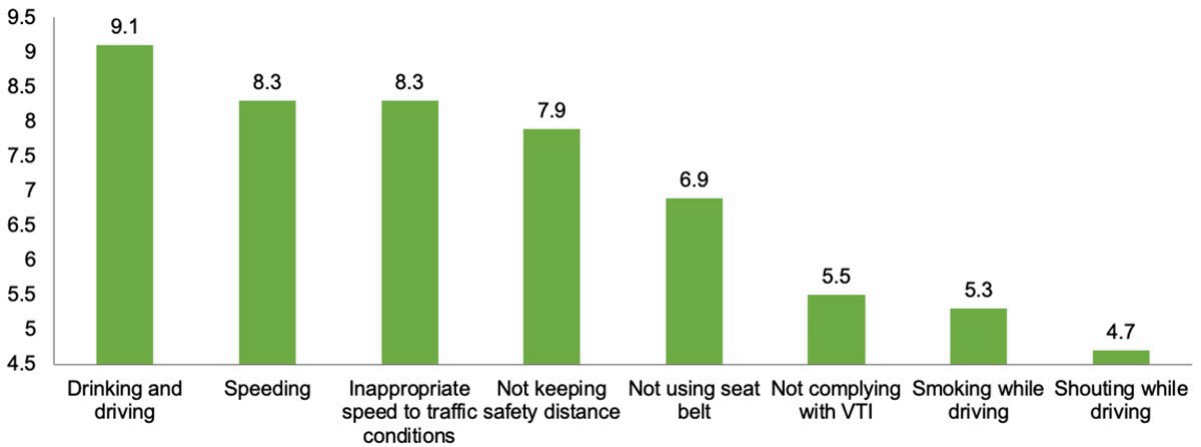
Perceived risk of having a road crash due to different driving misbehaviors.

As shown in Fig 4, drivers were also asked about the risk of having a road crash due to different driving misbehaviors. In this regard, driving with a vehicle that had not passed the VTI was evaluated as a moderate-low risk with an average score of 5.5 ± 2.8 out of 10 points. ANOVA results (*F*_(1,1074)_ = 10.99; *p*<0.05) showed that those drivers who have had a road crash reported significantly lower scores in risk perception of driving without VTI (*M* = 5.4; *SD* = 2.77) than those who had not (*M* = 5.7; *SD* = 2.86).

**Fig 5:**
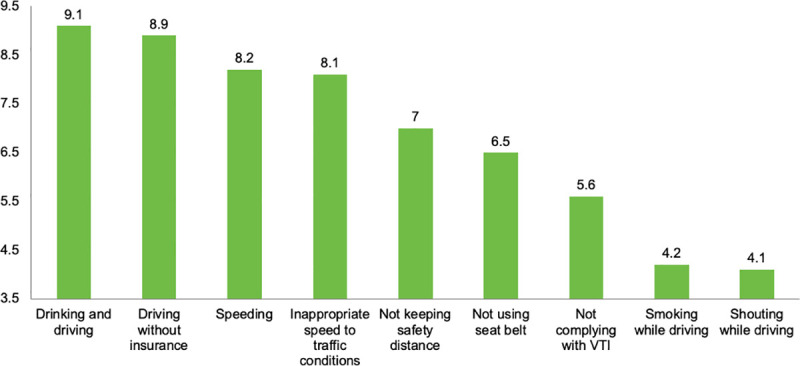
Perceived punishability of different driving misbehaviors.

As for the opinion of the respondents concerning the punishability of the aforementioned misbehaviors (*i*.*e*., the perceived likelihood of being sanctioned as a result of performing them), this outcome is graphically presented in Fig 5. Regarding the gender of the respondents (*F*_(1,1091)_ = 3.72; *p*<0.05), women showed significantly higher scores on the punishability of driving not complying with VTI’s standards (*M* = 5.8; *SD* = 2.76) than men (*M* = 5.5; *SD* = 2.87). In terms of road crashes’ history, drivers who had suffered a road crash obtained higher scores (*M* = 5.8; *SD* = 2.78) than those who had not (*M* = 5.4; *SD* = 2.89), being this difference statistically significant (*F*_(1,1091)_ = 4.41; *p*<0.05).

**Fig 6:**
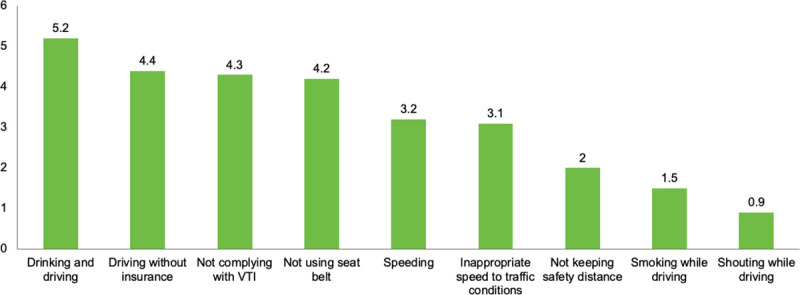
Perceived risk of being sanctioned for different road misbehaviors.

The perceived probability of being sanctioned out of 10 times of carrying out the aforementioned behaviors is presented in Fig 6. In this regard, driving with an outdated VTI certificate obtained third place in the probability of getting a penalty scale with an average score of 4.3 ± 2.6 out of 10 points.

## Discussion

Bearing in mind the central target of this research on the compliance of VTI’s standards, it is worth pointing out that legal regulation, including traffic rules and penalties, seems to affect drivers’ self-reported behaviors. Most of the drivers of our sample reported always driven complying with VTI’s standards. These results are in agreement with those published by Alonso [17] on compliance with VTI’s standards. 51.4% of the sample reported respecting the rules and being in fear of receiving a sanction as the main reasons to comply. In this regard, the most chosen reason to not comply was not realizing they had to take their vehicle to the inspection. These results are in concordance with those published by some authors on the public awareness of the traffic laws and the role of the state administration on this behavior [7, 8]. In this line, one questionnaire-based study reported a moderate-to-high degree of knowledge of road traffic regulation, forgetfulness being the main cause of not complying with the law [5, 28].

Delving into the habits and attitudes towards VTI’s standards, most drivers identified driving with no VTI as a sanctionable behavior, the economic penalties being the most popular type of sanction as previously reported by other authors [17, 29]. This seems logical, taking into account the observed features of not realizing having an outdated VTI certificate as the most reported motive to not comply. Also, the mainly low-risk perception of having a road crash due to the maintenance of the vehicle and the low perceived punishability of driving with an outdated VTI are factors that led to choosing the economic penalties. Thus, both prison and administrative sanctions such as the loss of the driving license are assessed by drivers as less relevant punishment alternatives for this behavior. However, there is no agreement on the harshness of the penalties for those who had been sanctioned due to driving with no VTI. Indeed, the punishment the drivers received due to driving with an outdated VTI certificate appears to be quite effective, with most of them not driving again without VTI. This finding adds important information to previous questionnaire-based studies on which subjects who reported low effectivity on traffic penalties were those who had received a higher amount of penalties [17, 28].

Related to the main objective of the study, driving without VTI is a behavior miss-valued with a moderate to low risk, in the case of explaining a potential road crash. In contrast, based on the registered records, the proper performance of motor vehicle inspections has been associated, in several ways, with lower rates of accident (crash) mortality in terms of the potential risks both for the driver, passengers, and other road users [1, 9, 19]. In addition to this concern, participants who have had a road crash reported statistically significant (*p*<0.05) lower values. This result is in accordance with Chekijan et al. [7], who found out that drivers involved in a road crash were not more likely to comply with laws after the crash. In this sense, having accurate information on the risks involved in driving with an outdated VTI certificate and other misbehaviors that occurred while driving would be very convenient. The key to reducing this and other misbehaviors should be focused on the strengthening of driving responsibly as a part of road safety education [5, 13].

Driving not complying with VTI’s standards is also reported as one of the less punishable behaviors included in the questionnaire. However, participants perceived a high probability of being sanctioned due to carrying this behavior. In this way, the gender of the respondents and their previous history of road crashes arise as factors conditioning the answer, with women and drivers who had been involved in a road crash perceiving a statistically significant higher punishability (*p*<0.05). These are issues that should be addressed within the scope of the optimal application of laws to improve the compliance of drivers, passengers, and pedestrians with the traffic, as have been described in other studies dealing with the Spanish population [13] and others [5, 7, 8, 28].

Apart from the main objective of the study and considering that drivers’ misbehaviors greatly increase the risk of collisions [5, 9, 28, 30–32], it is worth highlighting that, in our results, driving after drinking alcohol is the most chosen one in terms of increasing the risk of crashing, punishability, and possibilities of being sanctioned. This result suggests that social campaigns are making effect, and people consider that driving after drinking alcohol can be a major cause of road crash [7, 9, 14–16]. However, surveys carried in other countries revealed a low awareness concerning alcohol and driving [15] and a low probability of being caught while drinking and driving [7].

In summary, the results of this research shed light on the driver’s awareness of complying or not complying with VTI’s standards and other risky behaviors. Most surveyed drivers self-reported always complying with the normative in this regard, the main reasons to comply being respecting the rules and being in fear of receiving a sanction. This fact, following other aforementioned authors [5, 8, 17, 28, 29, 33, 34], highlights the impact of policy and legislation on driver’s self-reported habits. Enforcement of laws that are consistent with the road reality continues to be essential in maintaining effectiveness and improving road safety. Achieving this requires the careful coordination of the matrix between community groups, educational and traffic institutions, politicians, road safety engineers, and automotive experts [7, 9, 15, 35].

### Limitations of the study and future research

This study used a considerably large and representative study sample, the statistical parameters and model fit coefficients were adequately verified, and the quality and value of the questionnaires had been previously supported by many empirical studies. However, some methodological and qualitative biasing sources should be considered. Firstly, the research was carried out by means of self-report-based data. Several studies have shown how self-report measures may carry different biases, such as acquiescent answers (i.e., the total agreement of participants with the presented questions), social desirability, and lack of sincerity, especially considering that most of the questionnaires were applied at the workplace, in the companies where the drivers were working. Furthermore, positive/negative affects/mood may impact the response style of participants, especially when addressing issues that may seem sensitive, such as occupational traffic crashes, even when responding to anonymous questionnaires, as pointed out by Chai et al. [36] and Af Wåhlberg [37] in previous studies dealing with drivers and their road safety outcomes. Also, some studies have documented the existence of substantial discordances between attitudes, self-reports, and observed behaviors of road users [8, 33].

Road safety regulations allow for the improvement of road users’ interactions, whatever their role is (e.g., pedestrians, passengers, drivers, cyclists). It is, therefore, necessary that all people are aware of these regulations. In this regard, it would be very useful to know which are the least known signs and rules in certain segments of the population. While this paper provides general knowledge about driver’s awareness of different driver’s risky behaviors, and more specifically on the compliment with VTI’s standards, further research should be addressed on filling the remaining gaps in the area of compliance with traffic normative. It would also be of great interest for the traffic authorities to find a profile of a driver who tends to not comply with such normative. This could be achieved through correlations and regression to be able to predict the dependent variable.

## Conclusion

This study analyzes the compliance with the VTI’s normative of a representative sample of Spanish drivers, and their attitudes towards some risky behaviors. Although driving without VTI is considered a low-risk behavior, most of the subjects declared complying with VTI’s standards. The main cause of not complying with VTI was forgetfulness, suggesting the need of strengthening drivers’ awareness in this matter. Traffic penalties seemed to have a positive effect on driver’s VTI-related conduct. The information presented in this paper could be of interest and very useful for traffic authorities, law enforcement agencies, and educational institutions designated to train drivers and other road users.

## Supporting information

S1 Appendix Template used by the interviewer. (DOCX)

S1 Dataset Raw data is available in the file (database) attached to the electronic version of this manuscript. (ZIP)
