# Supplementary material for: Compliance, practices, and attitudes towards VTIs (Vehicle Technical Inspections) in Spain: What prevents Spanish drivers from checking up their cars?
Source: PLoS One. 2021 Jul 19;16(7):e0254823. doi: 10.1371/journal.pone.0254823 (PMC8289058; doi:10.1371/journal.pone.0254823)
Supplement: S1 Appendix — (DOCX) [file pone.0254823.s001.docx]

**Vehicle technical inspection (VTI) and other driving behaviors**

**Date:**

**Study:**

**Technician:**

**Questionnaire:**

**Questionnaire:**

**Hello. I am an interviewer from a company dedicated to market studies called GFK. On this occasion, we are carrying out a study focused on road safety for the Research Institute on Traffic and Road Safety of the University of Valencia. Your opinion is vital for us, and thus, we would appreciate you answering a few questions. It will take no more than 10 minutes.**

**All the information will be strictly anonymized as gathered in the current law of data protection. Under no circumstance, your answers could be used against you. We beg for sincere answers. Thank you very much for your cooperation.**

**Driving experience**

Do you have a driving license?

- Yes 1
- No 2→End interview.

Sex:

- Female 1
- Male 2

Could you please tell me your age?

Autonomous community:

- Specify

Have you ever had a road crash? Being you, the person causing the crash or not.

- Yes 1
- No 2

**SPECIFIC QUESTIONS ABOUT ROAD RISKY BEHAVIORS**

Now you are going to hear several questions about your perception of some behaviors on the road.

BEHAVIOR 1 (Speeding above the legal limits)

Evaluate between 0 and 10 the risk you perceive in speeding as a cause of road crashes, being 0 minimum risk and 10 maximum risk.

| 0 | 1 | 2 | 3 | 4 | 5 | 6 | 7 | 8 | 9 | 10 |
| --- | --- | --- | --- | --- | --- | --- | --- | --- | --- | --- |
|  |  |  |  |  |  |  |  |  |  |  |

Between 0 and 10, please, evaluate whether speeding should be punished, being 0 no agreement and 10, maximum agreement.

| 0 | 1 | 2 | 3 | 4 | 5 | 6 | 7 | 8 | 9 | 10 |
| --- | --- | --- | --- | --- | --- | --- | --- | --- | --- | --- |
|  |  |  |  |  |  |  |  |  |  |  |

Out of 10 occasions of speeding, how many would you say are punished?

| 0 | 1 | 2 | 3 | 4 | 5 | 6 | 7 | 8 | 9 | 10 |
| --- | --- | --- | --- | --- | --- | --- | --- | --- | --- | --- |
|  |  |  |  |  |  |  |  |  |  |  |

BEHAVIOR 2 (Inappropriate speed to traffic conditions)

Evaluate between 0 and 10 the risk you perceive in driving at an inappropriate speed to traffic conditions as a cause of road crashes, being 0 minimum risk and 10, maximum risk.

| 0 | 1 | 2 | 3 | 4 | 5 | 6 | 7 | 8 | 9 | 10 |
| --- | --- | --- | --- | --- | --- | --- | --- | --- | --- | --- |
|  |  |  |  |  |  |  |  |  |  |  |

Between 0 and 10, please, evaluate whether driving at an inappropriate speed to traffic conditions should be punished, being 0 no agreement and 10, maximum agreement.

| 0 | 1 | 2 | 3 | 4 | 5 | 6 | 7 | 8 | 9 | 10 |
| --- | --- | --- | --- | --- | --- | --- | --- | --- | --- | --- |
|  |  |  |  |  |  |  |  |  |  |  |

Out of 10 occasions of driving at an inappropriate speed to traffic conditions, how many would you say are punished?

| 0 | 1 | 2 | 3 | 4 | 5 | 6 | 7 | 8 | 9 | 10 |
| --- | --- | --- | --- | --- | --- | --- | --- | --- | --- | --- |
|  |  |  |  |  |  |  |  |  |  |  |

BEHAVIOR 3 (Not keeping the safety distance)

Evaluate between 0 and 10 the risk you perceive in not keeping the safety distance as a cause of road crashes, being 0 minimum risk and 10, maximum risk.

| 0 | 1 | 2 | 3 | 4 | 5 | 6 | 7 | 8 | 9 | 10 |
| --- | --- | --- | --- | --- | --- | --- | --- | --- | --- | --- |
|  |  |  |  |  |  |  |  |  |  |  |

Between 0 and 10, please, evaluate whether not keeping the safety distance should be punished, being 0 no agreement and 10, maximum agreement.

| 0 | 1 | 2 | 3 | 4 | 5 | 6 | 7 | 8 | 9 | 10 |
| --- | --- | --- | --- | --- | --- | --- | --- | --- | --- | --- |
|  |  |  |  |  |  |  |  |  |  |  |

Out of 10 occasions of not keeping the safety distance, how many would you say are punished?

| 0 | 1 | 2 | 3 | 4 | 5 | 6 | 7 | 8 | 9 | 10 |
| --- | --- | --- | --- | --- | --- | --- | --- | --- | --- | --- |
|  |  |  |  |  |  |  |  |  |  |  |

BEHAVIOR 4 (Shouting or verbally insulting while driving)

Evaluate between 0 and 10 the risk you perceive in shouting or verbally insulting while driving as a cause of road crashes, being 0 minimum risk and 10, maximum risk.

| 0 | 1 | 2 | 3 | 4 | 5 | 6 | 7 | 8 | 9 | 10 |
| --- | --- | --- | --- | --- | --- | --- | --- | --- | --- | --- |
|  |  |  |  |  |  |  |  |  |  |  |

Between 0 and 10, please, evaluate whether shouting or verbally insulting while driving should be punished, being 0 no agreement and 10, maximum agreement.

| 0 | 1 | 2 | 3 | 4 | 5 | 6 | 7 | 8 | 9 | 10 |
| --- | --- | --- | --- | --- | --- | --- | --- | --- | --- | --- |
|  |  |  |  |  |  |  |  |  |  |  |

Out of 10 occasions of shouting or verbally insulting while driving, how many would you say are punished?

| 0 | 1 | 2 | 3 | 4 | 5 | 6 | 7 | 8 | 9 | 10 |
| --- | --- | --- | --- | --- | --- | --- | --- | --- | --- | --- |
|  |  |  |  |  |  |  |  |  |  |  |

BEHAVIOR 5 (Driving after drinking alcohol)

Evaluate between 0 and 10 the risk you perceive in driving after drinking alcohol as a cause of road crashes, being 0 minimum risk and 10, maximum risk.

| 0 | 1 | 2 | 3 | 4 | 5 | 6 | 7 | 8 | 9 | 10 |
| --- | --- | --- | --- | --- | --- | --- | --- | --- | --- | --- |
|  |  |  |  |  |  |  |  |  |  |  |

Between 0 and 10, please, evaluate whether driving after drinking alcohol should be punished, being 0 no agreement and 10, maximum agreement.

| 0 | 1 | 2 | 3 | 4 | 5 | 6 | 7 | 8 | 9 | 10 |
| --- | --- | --- | --- | --- | --- | --- | --- | --- | --- | --- |
|  |  |  |  |  |  |  |  |  |  |  |

Out of 10 occasions of driving after drinking alcohol, how many would you say are punished?

| 0 | 1 | 2 | 3 | 4 | 5 | 6 | 7 | 8 | 9 | 10 |
| --- | --- | --- | --- | --- | --- | --- | --- | --- | --- | --- |
|  |  |  |  |  |  |  |  |  |  |  |

BEHAVIOR 6 (Not using the seat belt)

Evaluate between 0 and 10 the risk you perceive in not using the seat belt as a cause of injuries in case of suffering a road crash, being 0 minimum risk and 10, maximum risk.

| 0 | 1 | 2 | 3 | 4 | 5 | 6 | 7 | 8 | 9 | 10 |
| --- | --- | --- | --- | --- | --- | --- | --- | --- | --- | --- |
|  |  |  |  |  |  |  |  |  |  |  |

Between 0 and 10, please, evaluate whether not using the seat belt should be punished, being 0 no agreement and 10, maximum agreement.

| 0 | 1 | 2 | 3 | 4 | 5 | 6 | 7 | 8 | 9 | 10 |
| --- | --- | --- | --- | --- | --- | --- | --- | --- | --- | --- |
|  |  |  |  |  |  |  |  |  |  |  |

Out of 10 occasions of not using the seat belt, how many would you say are punished?

| 0 | 1 | 2 | 3 | 4 | 5 | 6 | 7 | 8 | 9 | 10 |
| --- | --- | --- | --- | --- | --- | --- | --- | --- | --- | --- |
|  |  |  |  |  |  |  |  |  |  |  |

BEHAVIOR 7 (Smoking while driving)

Evaluate between 0 and 10 the risk you perceive in smoking while driving as a cause of road crashes, being 0 minimum risk and 10, maximum risk.

| 0 | 1 | 2 | 3 | 4 | 5 | 6 | 7 | 8 | 9 | 10 |
| --- | --- | --- | --- | --- | --- | --- | --- | --- | --- | --- |
|  |  |  |  |  |  |  |  |  |  |  |

Between 0 and 10, please, evaluate whether smoking while driving should be punished, being 0 no agreement and 10, maximum agreement.

| 0 | 1 | 2 | 3 | 4 | 5 | 6 | 7 | 8 | 9 | 10 |
| --- | --- | --- | --- | --- | --- | --- | --- | --- | --- | --- |
|  |  |  |  |  |  |  |  |  |  |  |

Out of 10 occasions smoking while driving, how many would you say are punished?

| 0 | 1 | 2 | 3 | 4 | 5 | 6 | 7 | 8 | 9 | 10 |
| --- | --- | --- | --- | --- | --- | --- | --- | --- | --- | --- |
|  |  |  |  |  |  |  |  |  |  |  |

BEHAVIOR 8 (Driving without insurance)

Evaluate between 0 and 10 the risk you perceive in driving without insurance as a cause of legal conflicts in case of suffering a road crash, being 0 minimum risk and 10, maximum risk.

| 0 | 1 | 2 | 3 | 4 | 5 | 6 | 7 | 8 | 9 | 10 |
| --- | --- | --- | --- | --- | --- | --- | --- | --- | --- | --- |
|  |  |  |  |  |  |  |  |  |  |  |

Between 0 and 10, please, evaluate whether driving without insurance should be punished, being 0 no agreement and 10, maximum agreement.

| 0 | 1 | 2 | 3 | 4 | 5 | 6 | 7 | 8 | 9 | 10 |
| --- | --- | --- | --- | --- | --- | --- | --- | --- | --- | --- |
|  |  |  |  |  |  |  |  |  |  |  |

Out of 10 occasions of driving without insurance, how many would you say are punished?

| 0 | 1 | 2 | 3 | 4 | 5 | 6 | 7 | 8 | 9 | 10 |
| --- | --- | --- | --- | --- | --- | --- | --- | --- | --- | --- |
|  |  |  |  |  |  |  |  |  |  |  |

**SPECIFIC QUESTIONS ABOUT THE BEHAVIOR TOWARDS VTI**

Have you ever been fined due to driving with a non-valid VTI?

- YES 1
- NO 2

If your answer was yes, which are the reasons to not comply with VTI’s standards?

**Specify:**

If your answer was no, which are the reasons to comply with the VTI’s standards?

**Specify:**

Evaluate between 0 and 10 the risk you perceive in driving without a valid VTI as a cause of road crashes, being 0 minimum risk and 10, maximum risk.

| 0 | 1 | 2 | 3 | 4 | 5 | 6 | 7 | 8 | 9 | 10 |
| --- | --- | --- | --- | --- | --- | --- | --- | --- | --- | --- |
|  |  |  |  |  |  |  |  |  |  |  |

Between 0 and 10, please, evaluate whether driving without a valid VTI should be punished, being 0 no agreement and 10, maximum agreement.

| 0 | 1 | 2 | 3 | 4 | 5 | 6 | 7 | 8 | 9 | 10 |
| --- | --- | --- | --- | --- | --- | --- | --- | --- | --- | --- |
|  |  |  |  |  |  |  |  |  |  |  |

Is driving without a valid VTI punishable?

- YES 1
- NO 2

Out of 10 occasions of driving without a valid VTI, how many would you say are punished?

| 0 | 1 | 2 | 3 | 4 | 5 | 6 | 7 | 8 | 9 | 10 |
| --- | --- | --- | --- | --- | --- | --- | --- | --- | --- | --- |
|  |  |  |  |  |  |  |  |  |  |  |

In your opinion, the sanctions due to driving with a non-valid VTI should be:

*(Read each of the options)*

|  | Economic penalties | Prison | Temporary or permanent suspension of the driving license |
| --- | --- | --- | --- |
| YES |  |  |  |
| NO |  |  |  |

If you have been sanctioned due to driving with a non-valid VTI, please evaluate the harshness of this sanction:

- Excessive 1
- Adequate 2
- Scarce 3

If you have been fined due to driving with an outdated VTI, does this fact modified your behavior in this sense?

- YES 1
- NO 2

DATOS DE CLASIFICACIÓN SOCIAL

**PLACE:**

**PHONE NUMBER:**

**INTERVIEW TIME: DATE:**

**INTERVIEWER:**

“Carried out under the code of conduct of ESOMAR”

SIGNED:
